# Cluster randomised controlled trial of behavioural intervention program: a study protocol for control of hypertension among teachers in schools in Kerala (CHATS-K), India

**DOI:** 10.1186/s12889-019-8082-5

**Published:** 2019-12-21

**Authors:** G. K. Mini, P. S. Sarma, K. R. Thankappan

**Affiliations:** 1 Achutha Menon Centre for Health Science Studies, Sree Chitra Tirunal Institute for Medical Sciences and Technology, Trivandrum, Kerala 695011 India; 2 Global Institute of Public Health, Ananthapuri Hospitals and Research Institute, Trivandrum, Kerala 695024 India; 3 Women’s Social and Health Studies Foundation, Trivandrum, Kerala 695029 India; 4 Department of Public Health and Community Medicine, Central University Kerala, Kasaragod, Tejaswini Hills, Periye, Kerala 671320 India

**Keywords:** Hypertension, Control of hypertension, Self-management, Intervention, School teachers, Kerala, India

## Abstract

**Background:**

Control of blood pressure among hypertensives is a major challenge around the world. Interventions for improving hypertension control in India are very limited. This paper describes the protocol for a cluster randomized controlled trial of efficacy of behavioural intervention on control of hypertension among school teachers in Kerala.

**Methods:**

A total of 92 schools are randomised to intervention and control group in Kerala. A baseline survey was conducted in all schools to assess the prevalence of hypertension and its risk factors among school teachers in Thiruvananthapuram district of Kerala state, India. Teachers in both sets of schools will receive a leaflet containing details on the importance of controlling hypertension. With the objective of improving control of hypertension, the intervention schools will additionally receive self-management education and behavioural intervention programs delivered by trained intervention managers along with measurement of weight, waist circumference and blood pressure. This intervention program will be developed based on the findings of the baseline survey and selected components of successful models of hypertension control from previous research done in similar settings. The intervention will be given for 3 months after which a post-survey will be conducted among teachers of both control and intervention schools. The primary outcome is change in control of hypertension and secondary outcome is the change in behavioural risk factors of hypertension both in the control and intervention groups.

**Discussion:**

This is the first comprehensive study looking at the efficacy of behavioural intervention on hypertension control among school teachers in Kerala, India. This study is likely to provide an upper estimate of behavioural intervention on hypertension control since teachers are reported to have one of the highest compliance rates of behavioural intervention.

**Trial registration:**

This trial was prospectively registered with the Clinical Trials Registry of India [CTRI/2018/01/011402] on 18 January 2018.

## Background

Hypertension is the major contributor to the global burden of diseases and mortality [1]. It is the most important Noncommunicable Disease (NCD) risk factor in the world as well as in India [2]. Blood pressure control among hypertensives is a major challenge around the world [3]. A comparative analysis of national surveys in 20 countries on hypertension control reported that in various parts of the world, hypertension is not adequately controlled with medication [4]. There are wide disparities in control of hypertension globally. In 2010, worldwide only 13.8% had their blood pressure controlled which was higher for high-income countries (28.4%) compared to low-income countries (7.7%) [5] In this systematic review, the United States had the highest hypertension control rate (men:48.3%, women:54.1%) whereas the corresponding figure for India was 7.3 and 7.8%. Uncontrolled hypertension is associated with high risk for development of heart disease, stroke, chronic kidney disease, retinopathy and peripheral vascular disease [6]. Implementation of innovative and cost-effective programs for control of hypertension is a major public health challenge, particularly in developing countries [5]. This could be the reason why interventions to control hypertension are limited in developing countries such as India. One such study from the Kerala state of India, the most advanced state in epidemiological transition [7], reported a four-fold increase in control of hypertension after intervention among adult hypertensives in a rural community [8]. This seven-year follow-up study reported that health promotion messages for tobacco cessation, reducing alcohol consumption, healthy eating, and physical activity was effective in control of hypertension.

Kerala state has also reported the highest prevalence of cardiovascular diseases in India [9]. Furthermore, community-based studies from Kerala reported a comparatively higher NCD risk factor prevalence [10]. Interventions among working population were found to be effective in modifying the NCD risk factors in the state [11] and other parts of India [12]. In a study from rural Kerala, 24.4% of hypertensives were aware of their condition, 19.7% were treated, and only 6.4% had their blood pressure under control [13]. The control rate is very low compared to that of high-income countries [5].Recent reports from Kerala indicated that only 13% of the hypertensive population achieved adequate control of blood pressure [systolic blood pressure (SBP) < 140 mm of Hg and diastolic blood pressure (DBP) < 90 among hypertensives] [14]. A systematic review and meta-analysis of hypertension in Indian states indicated that different states in India showed different levels of control of hypertension [15].

Teachers are role models for the community and can play a major role in school-based health education programs [16–18]. An intervention study among school teachers was reported to be effective in tobacco control in the Indian state of Bihar [19]. They can also contribute to workplace excellence and community development in society. In a highly educated state like Kerala, where access to health care is one of the best in India, there is still a lack of effective interventions for improving awareness, treatment and control of hypertension. Recent findings of a randomized controlled trial from Nepal indicated a positive effect of lifestyle intervention along with monitoring of blood pressure on reduction of blood pressure in individuals with hypertension [20]. There are many randomised controlled trials (RCT) on the effect of interventions on prevention and lowering blood pressure in India [21, 22]. A community-based cluster randomised controlled trial to improve the control of hypertension in rural India has been initiated, the outcome of which is not yet published [23]. Control of Hypertension Among Teachers in Schools in Kerala (CHATS-K) is the first comprehensive trial to evaluate the efficacy of behavioural intervention on control of hypertension among school teachers in the state. This research protocol describes the baseline survey and the plan of intervention and post-intervention study details.

## Method/design

### Study design

The study is a cluster randomised controlled trial with schools as unit of randomisation. First a baseline survey was conducted for identifying those with hypertension. After the baseline survey, the total number of schools will be randomised into two equal groups: control and intervention schools. In the intervention schools, self-management education and behavioural intervention programs will be delivered by trained intervention managers along with the measurement of weight, waist circumference and blood pressure. After 3 months of intervention, we will evaluate the control of hypertension in intervention and control groups along with other risk factors of hypertension. The study is approved by the institute ethics committee of Sree Chitra Thirunal Institute of Medical Sciences and Technology, Trivandrum, India (Reg. No 799/2015).

### Setting

The study will be conducted in the state of Kerala, which is the most advanced Indian state in terms of both epidemiological and demographic transition [7]. In Kerala, Thiruvananthapuram district was selected based on its human development index which is similar to that of the state [24] and monitoring is feasible due to its proximity to the collaborating institute.

### Participants-inclusion and exclusion criteria

Eligible schools were government or government-aided schools in Thiruvananthapuram district of Kerala state in India. Eligible participants comprised consenting male and female teachers of the selected schools, aged 30–55 years. Participants who have a fluent understanding of spoken *Malayalam* (local language) and are willing to participate in the intervention program were included. We selected teachers who have a high probability that they would be in the same school for the next year. Participant will be excluded if they do not meet any of these criteria.

### Study sample

**Fig. 1:**
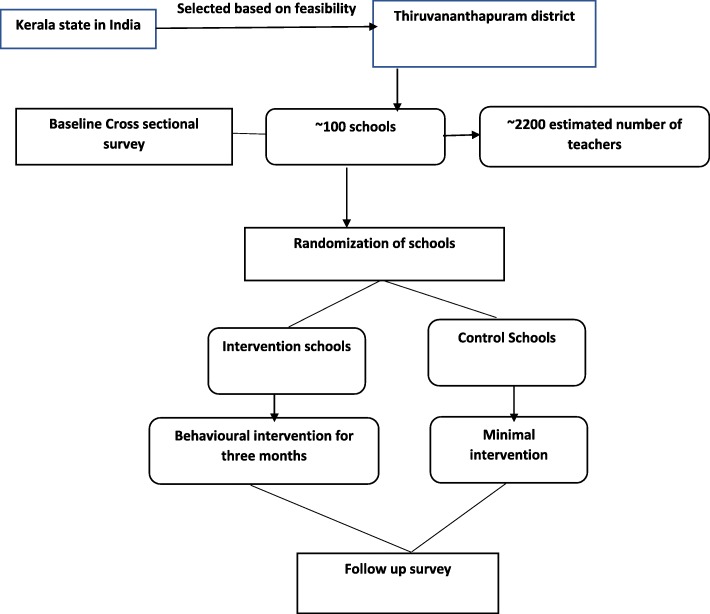
Outline of study design: enrolment, randomisation and follow up of study participants

Government or government-aided schools in Thiruvananthapuram district were identified as the primary sampling unit. The sample size for the baseline survey was 2200 teachers, expecting the prevalence of hypertension as 30% [14] to get a 95% confidence interval with an absolute precision of 2.5%, for an assumed design effect of 1.5 and non-response rate of 10%. The sample size was calculated using the Open Epi software. Among the total of 999 schools in the revenue district of Thiruvananthapuram, 902 are government or government-aided private schools. Of the 902 schools, we randomly selected 92 schools for the baseline survey using a computer-generated random assignment to get 2200 school teachers for the baseline survey (see Fig. 1). From each chosen school, we selected 15 teachers randomly if there were 15–55 teachers and for every additional 50 teachers, we selected another15 teachers. For schools with < 15 teachers, all teachers were selected. So, each cluster has approximately 15 teachers. The schools will be identified for the intervention and control by stratified randomisation with the number of hypertensives as the stratification variable.

### Baseline cross-sectional survey

A cross-sectional survey was conducted to identify school teachers with hypertension. A meeting was held with the education department, Government of Kerala to explain the study details. Office of the deputy director of education, Government of Kerala permitted to do the study and informed all the government and government-aided private schools in the selected district about the study. We then approached each of the selected school heads to get permission. From each school, teachers were selected randomly. Selected teachers were surveyed using a team of trained data collectors supervised by the research team. A brief on the study details were outlined verbally and in written format in *Malayalam* to the selected teachers before the initiation of the baseline survey. Informed written consent was obtained before the interview and measurements.

Data collection was based on an interviewer-administered survey which took approximately 30 min to complete. Additionally, physical measurements took around 15 min to complete. All assessments and data forms were checked on the day of completion. Any forms with missing data or inconsistencies were returned to the data collector for completion. Edit checks were performed and data verified as necessary.

We collected data (behavioural and anthropometric) for each participant in the selected schools using the World Health Organization (WHO) STEPS instrument for NCD risk factor surveillance. The validated standard WHO STEPS 2.1 version questionnaire was used. Baseline information on NCD behavioural risk factors was collected using WHO STEP-1, and physical measurements were done using WHO STEP-2 protocol. Blood Pressure was measured by OMRON BP apparatus (OMRON HEM-907, OMRON Healthcare Company, Kyoto, Japan). Height was measured using stadiometer, weight using SECA weighing machine and waist and hip circumference using constant tension tape using a standard protocol.

Besides, we asked questions on their morbidity pattern, cancer screening, perceptions on hypertension, opinion on the possible interventions which are likely to result in increasing control of hypertension and awareness of diabetes, hypertension and dyslipidemia status by asking questions on the history and treatment of the conditions. We also asked their knowledge about hypertension and its risk factors and their practices on healthy behaviour. The compliance to medication was also assessed using Hill-Born scale [25]. Their quality of life was assessed by the WHO quality of life questionnaire [26]. The main instruments and measurements chosen for this study are based on recommendations from the WHO STEPwise approach to NCD risk factor surveillance [27] to enable comparison of our findings to those of others.

### Outcome measurements

Prevalence, awareness, treatment and control of hypertension along with other risk factors of hypertension are collected during the baseline survey. The newly diagnosed hypertensives in the baseline survey were asked to visit their regular doctor or the nearest government primary health centre/hospital for confirmation of hypertension and management. The primary outcome of the study will be control of hypertension (defined as SBP < 140 mmHg and DBP < 90 mm [28]). This will be compared between intervention and control groups after 3 months of intervention. We will also compare the difference in mean systolic and mean diastolic blood pressure at baseline and post intervention period for control and intervention group. Secondary outcomes will be awareness and treatment rates for hypertension among teachers of intervention and control groups. We will also assess the change in risk factors of hypertension such as tobacco, alcohol, physical inactivity, unhealthy diet and, overweight/obesity after the intervention in the intervention group and the control group.

### Blinding

The allocation of sequence, enrolment of participants, and assignment of participants to interventions will be done by a senior statistician based at the implementing institute. The allocation of participants in the intervention group or the control group will be blinded to the outcome assessors. Since this is a behavioural intervention trial, blinding cannot be applied to the persons delivering the intervention.

### Plan for data collection and analysis

A follow-up survey will be conducted after the intervention to assess the change in control of hypertension, awareness and treatment for hypertension as well as other risk factors of hypertension both in control and intervention groups. Baseline survey data will be analysed to evaluate the prevalence of hypertension, and other behavioural risk factors of hypertension. Medication adherence will also be assessed. The quality of life will be analysed with hypertension status. Analysis of the opinion among school teachers regarding the feasibility of intervention options will also be done. Based on these results and already reported intervention programs in the state we will develop interventions suitable for school teachers on controlling hypertension. We will compare the control of hypertension in both study groups with the findings from baseline and post survey.

### Intervention components

Based on the findings of the baseline survey and findings from earlier research in the state, we will prepare the content of the intervention for improving the control of hypertension for school teachers. Also, selected component of successful models on risk factors of non-communicable diseases including hypertension, from previous research done in the state, which showed improvement in control of hypertension [8], will be incorporated into the intervention program. We will also take the relevant components from similar NCD prevention frameworks done in the state [14]. The intervention session will be designed with self-management education and research team supported intervention. Monitoring of blood pressure was reported to improve blood pressure control in a randomised trial among hypertensive patients in North Carolina [29]. Since we took the schools as the unit of randomisation, all teachers in the intervention schools will receive the intervention. We have gathered details from selected schools to know the feasibility of the intervention to improve hypertension control. Based on these findings, we will design a workable intervention program for school teachers in this community.

A multi-component intervention program will be implemented in intervention schools. Teachers in the intervention schools will receive educational intervention on lifestyle modification including healthy diet habits, importance of physical activity, tobacco and alcohol cessation programs and awareness, control and treatment of hypertension and the importance of medication adherence on hypertension. During the intervention period, all the schools in the intervention group will receive the above education components once every 2 weeks for a period of 3 months. Thus, each school will receive six such sessions. In these sessions, educational materials including healthy lifestyle practices for hypertension control will be given to all teachers in the intervention group. Health education classes including power point presentation, educational videos and interactive discussions will be part of these sessions at schools. All these sessions will be given as a group activity in each of the intervention schools. During these sessions, attention will also be given to the pre-hypertensives identified in the baseline survey. This would help to reduce the progression rate from pre-hypertension to hypertension and is also likely to reduce the incidence of hypertension.

In each of these meetings, weight and waist circumference will be measured along with blood pressure for all teachers who participated in the baseline survey and the values will be communicated to them. Questions on medication for hypertension and health care visit for high blood pressure during the last 2 weeks will also be asked to hypertensive teachers and responses will be noted. These intervention sessions will be face-to-face with the objective to motivate school teachers to increase the control rate of hypertension. During the intervention period, the development of changes in other risk factor behavioural characteristics will be monitored, and the sessions will help to overcome barriers and difficulties to control hypertension. Trained health educators, researchers and public health experts will handle the intervention components.

A WhatsApp group will be formed for those with hypertension identified during the baseline survey, and relevant educational materials and information will be regularly shared through the WhatsApp message system. Regular short message service (SMS) will be sent to those in the hypertension group as a reminder to take medication and keep healthy lifestyle practices for control of hypertension. Regular monitoring of blood pressure will be insisted on by messages through this smart-phone based intervention. The fidelity of the WhatsApp intervention will be ensured by contacting a sample of the participants over phone and finding out whether they received the Whatsapp message and if they received what was the message and what did they do about that. The intervention will be delivered for 3 months.

### Control group

During the baseline survey, all teachers who participated in this study are informed about their blood pressure, weight, height and waist circumference measurements. Those who are identified as hypertensives (SBP > =140 mmHg or DBP > =90 mmHg) during the survey are advised to check their blood pressure again for further confirmation and management. At the end of the baseline survey, all teachers participated in this study are provided educational material on hypertension and its risk factors. No other intervention will be given to the control group during the intervention period.

### Data management and statistical analysis

All assessments and data forms will be completed in real time on the day of measurement. Edit checks will be performed and data verified as necessary. Access to data will be restricted to the leading researchers. Hard copies of data will be stored in a secure place of storage while soft data will be stored securely using file locks and passwords. All identifiable information will be stored in a separate database from the other data. Data will be stored for 5 years after completion of the project. We will use intention-to-treat analysis for comparing the change in control of hypertension both among intervention and control groups.

### Trial registration

This trial was registered with the Clinical Trials Registry of India (CTRI) prospectively on 18th January 2018 [CTRI/2018/01/011402]. We started recruiting participants after the registration number has been received.

### Dissemination

Each participant is assigned a unique identification number after the baseline survey. Opportunities for active dissemination of findings will be pursued throughout the study by submission for presentation at national and international conferences and through publication in peer-reviewed national and international journals. The overall study results will be disseminated to participating schools. The details of the intervention program will also be communicated through publications to understand the details of the efficacy trial of control of hypertension among school teachers. After the post-survey, the comparative findings on control of hypertension will be disseminated for both control and intervention group. The funding agency will also be informed of the findings of the study. Findings will also be shared with the Department of Education, Government of Kerala.

## Discussion

Comprehensive estimation of the efficacy of the behavioural intervention for control of hypertension among school teachers is the first of its kind in India. Healthy teachers provide continuity and stability so essential for educational success. Also, Kerala state in India is having the highest level of education and has reported a high prevalence of hypertension [10]. The findings of the present study are likely to be an upper estimate of the level of efficacy of the behavioural intervention in control of hypertension in India. A short intervention period of 3months was a limitation of the study. However similar short intervention studies to improve hypertension control was reported earlier [23].

There is limited evidence on the efficacy of educational intervention with regular monitoring of blood pressure for improving control of hypertension in India. The intervention will improve the awareness of hypertension control and other risk factors like tobacco use, alcohol consumption, physical inactivity and unhealthy diet. It also brings awareness of the importance of taking regular medication for controlling hypertension. This intervention program will incorporate the previous experience of hypertension control programs conducted in similar settings [8]. We have done a formative research among school teachers to assess the possible components of intervention. The program is expected to increase their awareness on lifestyle risk factors which are also beneficial to the community irrespective of the trial outcome, which is another strength of the study. A meticulous randomisation process followed in this study is another strength which allows comparison, statistical reliability and minimises biases as well as confounding factors, which is the strongest possible analysis of the effect of an intervention. The randomisation followed in this study mitigates major operational biases.

In particular, we have shown that this intervention is applicable across very diverse rural settings, so there is considerable potential to implement and scale up across rural India and potentially other resource-poor regions in other countries. Future programs, incorporating comprehensive collection of data such as changes in medication use, changes to lifestyle behaviours, and knowledge of hypertension may help tease out which factors are best targeted to improve the control of hypertension in these settings, if successful, for scale up and implementation among similar groups and educational settings.

This study is likely to have an impact not only on teachers but also on students and their family members indirectly. If this intervention is found to be effective, the findings will provide a practical approach towards control of hypertension and other non-communicable diseases and their risk factors among school teachers in a similar setting. Also, comprehensive collection of data on behavioural life style changes in this study would help to prioritise the risk factors for control of hypertension. We also hypothesize that an intervention among school teachers would provide a higher level of impact of such an intervention program on control of hypertension. If such an intervention is successful among school teachers, it opens the way for scale up and implementation among similar groups and educational settings to achieve adequate control of hypertension in a cost-effective way. These findings will have important implications in the medical and public health field in controlling hypertension.

### Trial status

Protocol version number − 25 dated 27th March 2019.

The study began in January 2018. Baseline data collection was completed in July 2018. The intervention program is ongoing. A follow-up survey will be done and the final results are expected in August 2019.

## Data Availability

The datasets used and analysed during the current study are available from the corresponding author on reasonable request.

## Abbreviations

CHATS-K: Control of Hypertension Among Teachers in Schools in Kerala
CTRI: Clinical Trials Registry of India
DBP: diastolic blood pressure
NCD: Noncommunicable Disease
RCT: randomised controlled trials
SBP: systolic blood pressure
SMS: short message services
WHO: World Health Organization
